# Four different gene-related cone–rod dystrophy: clinical and genetic findings in six Chinese families with diverse modes of inheritance

**DOI:** 10.3389/fgene.2023.1157156

**Published:** 2023-11-02

**Authors:** Zhen Li, Wanyu Cheng, Feiyin Zi, Juan Wang, Xiaoyu Huang, Xunlun Sheng, Weining Rong

**Affiliations:** ^1^ Ningxia Eye Hospital, People’s Hospital of Ningxia Hui Autonomous Region, Third Clinical Medical College of Ningxia Medical University, Yinchuan, China; ^2^ Department of Ophthalmology, Qingdao West Coast New District Central Hospital, Qingdao, China; ^3^ Gansu Aier Optometry Hospital, Lanzhou, China

**Keywords:** cone–rod dystrophy, whole-exome sequencing, genotype, clinical phenotype, variant

## Abstract

**Purpose:** To investigate pathogenic variants in six families with cone–rod dystrophy (CORD) presenting various inheritance patterns by using whole-exome sequencing (WES) and analyzing phenotypic features.

**Methods:** A total of six families with CORD were enrolled in Ningxia Eye Hospital for this study. The probands and their family members received comprehensive ophthalmic examinations, and DNA was abstracted from patients and family members. Whole-exome sequencing was performed on probands to screen the causative variants, and all suspected pathogenic variants were determined via Sanger sequencing. Furthermore, co-segregation analysis was performed on available family members. The pathogenicity of novel variants was predicted using *in silico* analysis and evaluated according to the American College of Medical Genetics and Genomics (ACMG) guidelines.

**Results:** Of the six families, two families were assigned as X-linked recessive (XL), two families were assigned as autosomal recessive (AR), and two families were assigned as autosomal dominant (AD). Pathogenic variants were detected in *CACNA1F* in two X-linked recessive probands, among which family 1 had a hemizygous frameshift variant c.2201del (p.Val734Glyfs*17) and family 2 had a hemizygous missense variant c.245G>A (p.Arg82Gln). Both probands had high myopia, with fundus tessellation accompanied by abnormalities in the outer structure of the macular area. The homozygous splice variant c.2373 + 5G>T in *PROM1* and the homozygous nonsense variant c.604C>T (p.Arg202Ter) in *ADAM9* were detected in two autosomal recessive families of the probands. Both probands showed different degrees of atrophy in the macular area, and the lesions showed hypofluorescence changes in autofluorescence. The heterozygous variation in *CRX* c.682C>T (p.Gln228Ter) was detected in two autosomal dominant families. The onset age of the two probands was late, with better vision and severe macular atrophy. According to ACMG guidelines and the analysis of online *in silico* tools, all variations were labeled as potentially harmful or pathogenic.

**Conclusion:** Pathogenic variants in *CACNA1F*, *PROM1*, *ADAM9*, and *CRX* genes were identified in six families affected by the diverse inheritance patterns of CORD. Furthermore, the potential impact of the nonsense-mediated decay (NMD) mechanism on the manifestation of CORD phenotypes was examined and addressed. Simultaneously, the spectrum of pathogenic variants and clinical phenotypes associated with the CORD gene was extended.

## 1 Introduction

Cone–rod dystrophy (CORD) is a prevalent type of monogenic retinal disease observed in clinical settings. The condition is primarily characterized by atypical or predominantly atypical cone cell functionality, accompanied by various levels of atypical rod cell functionality. Its occurrence is estimated to be approximately 1 in 40,000 individuals (Thiadens et al., 2012). It usually presents as a progressive vision loss starting in adolescence or early adulthood, which may be accompanied by photophobia and varying degrees of color vision abnormalities (Boulanger-Scemama et al., 2019). In the advanced phases, certain individuals may exhibit nystagmus and experience a gradual decline in visual acuity, which may be accompanied by nyctalopia (Boulanger-Scemama et al., 2019).

The inheritance patterns of CORD include autosomal dominant (AD), autosomal recessive (AR), and X-linked recessive (XL) (Wawrocka et al., 2018). So far, 37 genes have been reported in the RetNet database associated with CORD (RetNet: https://sph.uth.edu/Retnet/sum-dis.htm), of which 10 genes were related to autosomal dominant [*AIPL1* (Sohocki et al., 1998), *CRX* (Sohocki et al., 1998), *GUCA1A* (Downes et al., 2001), *GUCY2D* (Kelsell et al., 1998), *PITPNM3* (Balciuniene et al., 1995), *PROM1* (Michaelides et al., 2003), *PRPH2* (Arikawa et al., 1992), *RIMS1* (Johnson et al., 2003), *SEMA4A* (Abid et al., 2006), and *UNC119* (Kobayashi et al., 2000)], 25 genes were related to autosomal recessive [*ABCA4* (Allikmets, 2000), *ADAM9* (Danciger et al., 2001), *ATF6* (Kohl et al., 2015), *C21orf2* (Abu-Safieh et al., 2013), *C8orf37* (van Huet et al., 2013), *CACNA2D4* (Wycisk et al., 2006), *CDHR1* (Ostergaard et al., 2010), *CEP78* (Nikopoulos et al., 2016), *CERKL* (Aleman et al., 2009), *CNGA3* (Wissinger et al., 1998), *CNGB3* (Michaelides et al., 2004), *CNNM4* (Downey et al., 2002), *DYNC2I2* (Solaguren-Beascoa et al., 2021), *GNAT2* (Aligianis et al., 2002), *IFT81* (Dharmat et al., 2017), *KCNV2* (Wu et al., 2006), *PDE6C* (Thiadens et al., 2009), *PDE6H* (Piri et al., 2005), *POC1B* (Durlu et al., 2014), *RAB28* (Roosing et al., 2013), *RAX2* (Wang et al., 2004), *RDH5* (Nakamura et al., 2000), *RPGRIP1* (Hameed et al., 2003), *SLC4A7* (Ahn et al., 2020), and *TTLL5* (Sergouniotis et al., 2014)], and two genes were related to X-linked patterns [*CACNA1F* (Jalkanen et al., 2003a) and *RPGR* (Yang et al., 2002)].

The earlier diagnosis of CORD mostly relied on clinical and familial history queries, multimodal imaging assessment, and electrophysiological testing (den Hollander et al., 2010). Due to its genetic complexity and clinical variability, CORD exhibits diverse clinical symptoms and signs that may vary throughout different phases. Furthermore, there exists phenotypic similarity between CORD and other inherited retinal diseases (Hamel, 2007). Therefore, accurately diagnosing a condition purely based on clinical presentations or genetic testing outcomes poses a challenge. The integration of genetic screening and clinical phenotypic analysis has the potential to enhance the efficacy and precision of clinical diagnosis in individuals affected by CORD (Birtel et al., 2018a). In this study, whole-exome sequencing (WES) was applied to detect variants in six CORD pedigrees with different inheritance modes, genotypes, and phenotypes; meanwhile, the results are presented.

## 2 Materials and methods

### 2.1 Ethical approval

The Ningxia Hui Autonomous Region People’s Hospital Ethics Committee granted approval for the study (approval number 20190909). All participants signed an informed consent form. All experiments were conducted in accordance with the Declaration of Helsinki.

### 2.2 Clinical data collection

Six CORD pedigrees were recruited from Ningxia Eye Hospital, People’s Hospital of Ningxia Hui Autonomous Region in 2021. The probands and members of their families underwent the required ophthalmologic examinations, including best-corrected visual acuity (BCVA), slit-lamp microscopy, chromoptometry (fifth version color imaginative and prescient examination plates, Ziping YU), dilated fundus examination with photographs (TRC-50DX, Topcon Inc.), optical coherence tomography (OCT, HDOCT4000, Carl Zeiss Meditec, United States), electroretinogram (ERG), and fundus autofluorescence (FAF).

### 2.3 Whole-exome sequencing

Peripheral venous blood (5 mL) samples were obtained from all participants for genomic DNA extraction using a QIAmp DNA Mini Blood Kit (Qiagen, Hilden, Germany). Whole-exome sequencing was performed on probands. The Agilent SureSelect exon capture kit was previously used to capture the exome. A high-throughput sequencer was once utilized to provide sequencing services (Illumina, HiSeq X Ten). Illumina base-calling software 1.7 was used to evaluate the raw sequencing data, which were then compared to the NCBI human genomic DNA reference sequence (NCBI construct 37.1). SOAP (http://soap.genomics.org.cn) and BWA software applications (http://bio-bwa.sourceforge.net/) were used to examine single-nucleotide variants (SNVs) and insertion and deletion editions (Indel). To reflect every variation present in the samples’ DNA sequences, co-segregation of the genotype and phenotype was previously established in ordinary household members, and Sanger validation was originally employed to remove false positives for potentially harmful variants. According to the genetic patterns of AD, AR, and XL, the family history was analyzed to establish their inheritance pattern.

### 2.4 *In silico* analysis

Standards and Guidelines for the Interpretation of Sequence Variants established in 2015 by the American College of Medical Genetics and Genomics (ACMG) were used to evaluate the pathogenicity of novel variations for genetic variation. The variant sites were filtered and screened by inserting the sites into the normal human databases, including the 1,000 genomes with normal population gene frequency, the Exome Aggregation Consortium (ExAC), and ExAC-EAS (approximately 4,000 East Asian data under ExAC). MAF <0.005 was used as a criterion to exclude benign variants. The gnomAD (all_gnomAD and eas_gnomAD) was used to analyze the frequency of variants in the normal population (and the normal East Asian population) of the gnomAD. Tools such as MutationTaster (http://mutationtaster.org/), PolyPhen-2 (http://genetics.bwh.harvard.edu/pph2/), SIFT (http://sift.jcvi.Org/www/SIFT.chr.coords.submit.html), CADD (https://cadd.gs.washington.edu/score), and REVEL (https://www.ncbi.nlm.nih.gov/pmc/articles/PMC5065685/) were used to predict the effects of variant sites on protein function. The measurements of the conservation of gene sequences across species in evolution were made using websites like GERP++ (https://bio.tools/gerp). If the projected score (HDIV/HVAR) of PolyPhen-2 is close to 1, then it is likely to be harmful (D); otherwise, it is either possibly damaging (P) or benign (B). The sequences underwent SIFT analysis, and amino acids that have probabilities below 0.05 are considered (D). The disease probability increases as the MutationTaster analysis score approaches 1, whose scores range from 0 to 1. REVEL is an ensemble score based on 13 individual scores for predicting the pathogenicity of missense variants, and its scores range from 0 to 1. The larger the score, the more likely the SNP to be damaging. The CADD raw score was used for the functional prediction of SNP. The larger the score, the more likely the SNP to be damaging. Its scores range from−6.458163 to 18.301497 in dbNSFP. The CADD PHRED-like score is a PHRED-like rank score based on whole-genome CADD raw scores. The larger the score, the more likely the SNP to be damaging. When considering the anticipated values for the conservativeness of the amino acid sequence, GERP++ values larger than 2 imply that the sequence is relatively conservative. The sequence is assumed to be highly conserved and deleterious if it has a high GERP score. When all predictions turned out to be accurate, variations were categorized as potentially pathogenic when used in conjunction with further data. Pathogenic variants were defined as frameshift, nonsense, and variants with the experimental proof of causing the loss of protein function. For the conservativeness study of variant loci, the online analysis tool MultAlin (http://sacs.ucsf.edu/cgi-bin/multalin.py) was employed. The spatial structures of these proteins were modeled using the freeware programs AlphaFold2 and Missense3D and then aligned using PyMOL 2.3.

## 3 Results

### 3.1 Family 1

**FIGURE 1:**
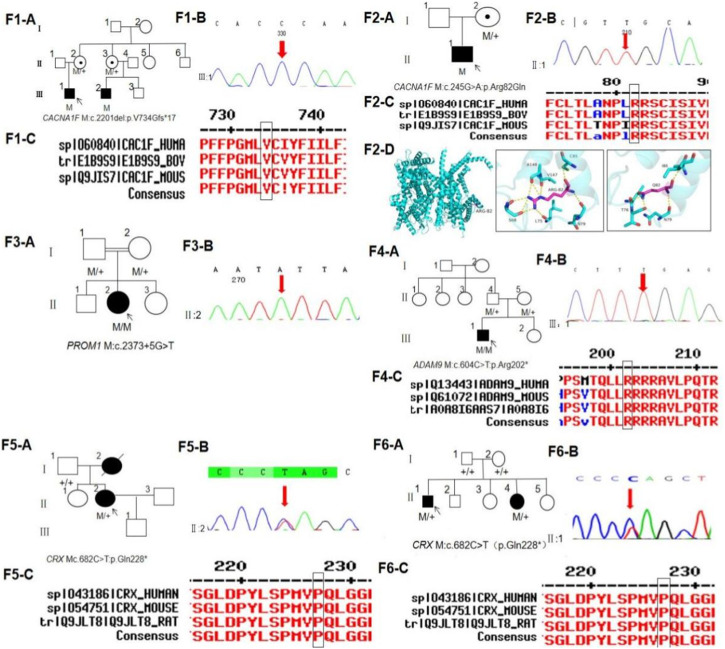
Pedigree, sequence analysis of six families with CORD. (F1-A) Pedigree of family 1. The filled black symbols represent the affected members, and the arrow denotes the proband. (F1-B) Sequence chromatograms of identified mutations in family 1. (F1-C) Homology of amino acid sequences between human *CACNA1F* and other species. The amino acid at position 734 is highly conserved among species. Mutated residue 734 is boxed and indicated. (F2-A) Pedigree of family 2. (F2-B) Sequence chromatograms of identified mutations in family 2. (F2-C) Homology of amino acid sequences between human *CACNA1F* and other species. The amino acid at position 82 is highly conserved among species. Mutated residue 82 is boxed and indicated. (F2-D) Homology model of the *CACNA1F* homeodomain (green) of family 2. Purple indicates the location of p.Arg82Gln in the protein structure. (F3-A) Pedigree of family 3. (F3-B) Sequence chromatograms of identified mutations in family 3. (F4-A) Pedigree of family 4. (F4-B) Sequence chromatograms of identified mutations in family 4. (F4-C) Homology of amino acid sequences between human *ADAM9* and other species. The amino acid at position 202 is highly conserved among species. Mutated residue 202 is boxed and indicated. (F5-A) Pedigree of family 5. (F5-B) Sequence chromatograms of identified mutations in family 5. (F5-C) Homology of amino acid sequences between human *CRX* and other species. The amino acid at position 228 is highly conserved among species. Mutated residue 202 is boxed and indicated. (F6-A) Pedigree of family 6. (F6-B) Sequence chromatograms of identified mutations in family 6. (F6-C) Homology of amino acid sequences between human *CRX* and other species.

**FIGURE 2:**
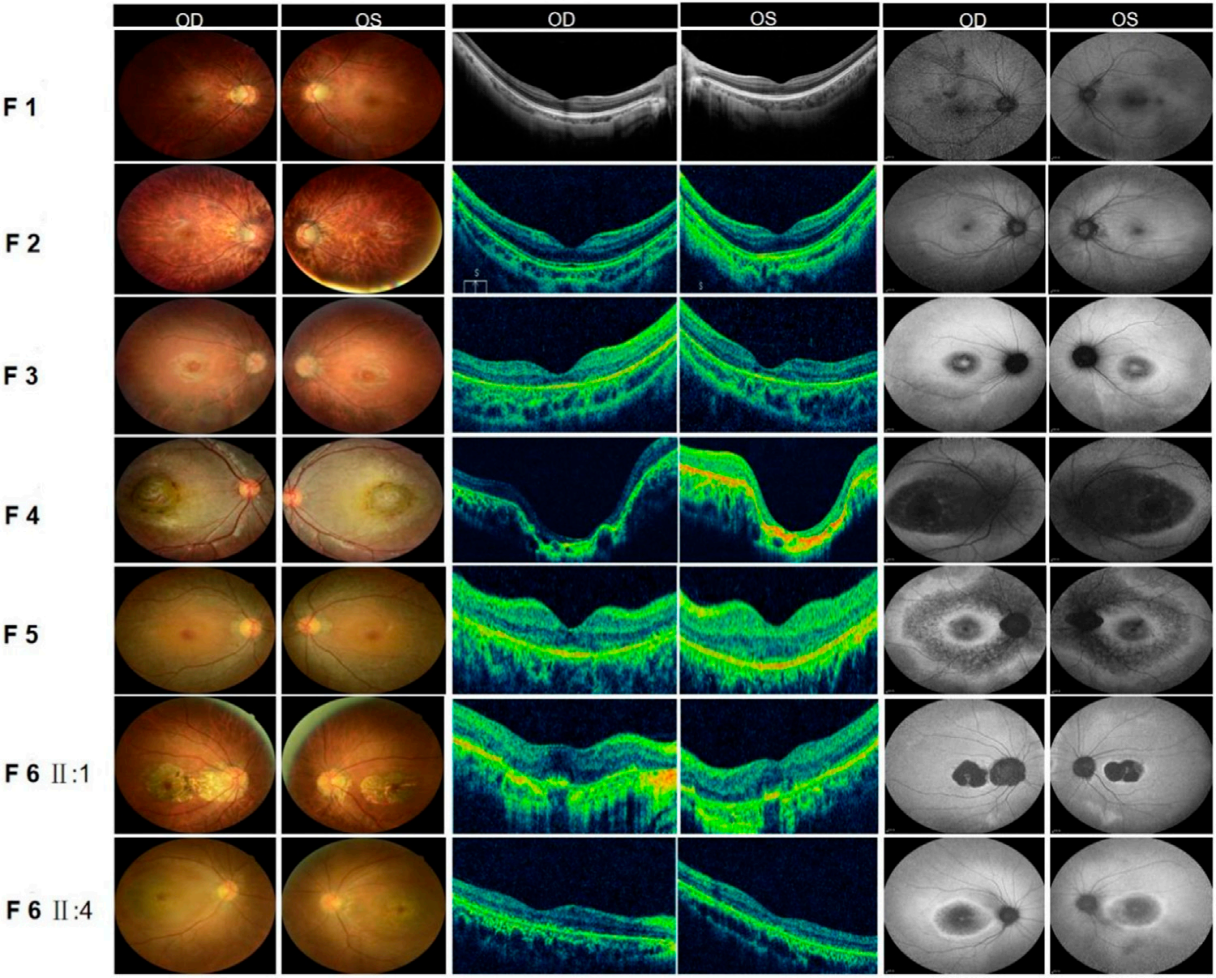
Fundus examination of six families with CORD. (F1) Fundus of both eyes in family 1: Fundus tessellation with no reflection was observed in the macular fovea; the peripheral retina did not show obvious bone-spicule pigmentation; OCT of the macula in both eyes suggested the disappearance of the light reflection signal in the chimeric zone of the fovea centralis; autofluorescence in both eyes suggested hypofluorescence in the macula. (F2) Fundus of both eyes in family 2: Fundus tessellation with macular atrophy was observed; the peripheral retina did not show obvious bone-spicule pigmentation; OCT of the macula in both eyes showed the thinning of the outer layer of the macula and the disappearance of the light reflection signal in the ellipsoid zone and chimeric zone of the fovea centralis; autofluorescence in both eyes suggested hypofluorescence in the macula. (F3) Fundus of both eyes in family 3: Fundus tessellation with an elliptic atrophy lesion was observed in the macular area and the peripheral retina did not show obvious bone-spicule pigmentation; OCT of the macula in both eyes showed significant atrophy and the thinning of the outer retina in the macula; autofluorescence in both eyes suggested that hyperfluorescence lesions were surrounded by low fluorescence in the macula of both eyes. (F4) Fundus of both eyes in family 4: Central retina with a macular coloboma-like lesion with a central atrophic scar surrounded by a ring of pigmentary changes, including a yellowish pigmentation of both eyes, was observed; OCT of the macula in both eyes showed the atrophic and ill-defined retinal layers, alterations and disruptions of the retinal pigment epithelium, and macular posterior staphyloma; autofluorescence showed hypofluorescence in the macula of both eyes. (F5) Fundus of both eyes in family 5: Elliptic atrophy lesion with no foveal reflection was observed in the macular area; OCT of the macula in both eyes showed atrophy of the outer retina and RPE layer in the macula; autofluorescence results of both eyes suggested that hyperfluorescence lesions were surrounded by low fluorescence in the macula of both eyes. (F6) Fundus of both eyes in family 6: Fundus photograph: The proband (II:1) and his sister (II: 4) all showed fundus tessellation with geographic atrophy in the macula; OCT: The proband (II:1) showed obvious thinning of central retinal thickness, and the outer nuclear layer and the ellipsoid zone disappeared in both eyes; OCT of his sister (II: 4) showed atrophy of the outer retina and RPE layer in the macula; FAF: The proband (II:1) and his sister (II: 4) all showed that hyperfluorescence lesions were surrounded by low fluorescence in the macula of both eyes.

The proband of family 1 was a 34-year-old man who complained of reduced vision in both eyes with photophobia for more than 10 years. He denied a consanguineous marriage history (Figure 1 F1-A). He had dyserythrochloropsia and BCVA of 0.4 in both eyes (Table 1). The anterior segment was normal, fundus tessellation with no reflection was observed in the macular fovea, and the peripheral retina did not show obvious bone-spicule pigmentation. OCT showed the disappearance of the light reflection signal in the chimeric zone of fovea centralis, and autofluorescence showed hypofluorescence in the macula of both eyes (Figure 2-F1). ERG recording revealed a mild decline in scotopic and severe or moderately reduced photopic responses (Table 1). The condition was diagnosed as CORD according to the clinical phenotype. The proband and his affected cousin carried a hemizygous frameshift variant c.2201del (p.Val734Glyfs*17) in *CACNA1F*, and Sanger sequencing showed that the unaffected mother and aunt carried the same heterozygous variant in *CACNA1F* (Table 2) (PP1_Supporting), suggesting the co-segregation of a genotype and clinical phenotype (Figure 1 F1-A), consistent with the mode of the X-linked recessive inheritance. The East Asian population (ExAC_EAS) and gnomAD databases and prior reports of the frameshift variant were negative (PM2_Moderate). The c.2201del variant led to a premature stop codon at position 17 of the new reading frame and caused a frameshift beginning with codon valine 734, changing this amino acid to a glycine residue (p.Val734Glyfs*17). Most of the proteins produced as a consequence of the truncated variants that were inactive or lost their normal function, which resulted in the premature termination of polypeptide chain synthesis (PVS1_Very Strong). It is anticipated that CACNA1F protein production (including truncated polypeptides) will be eliminated from mutant alleles by nonsense-mediated mRNA decay (NMD).Various bioinformatics computing software programs indicated the deleterious impact of this variant (PP3_Supporting). Moreover, proteome conservation analysis revealed that the amino acid at position 734 was substantially conserved across several taxa (Figure 1 F1-C), indicating that the variation at this location is more likely to have an impact on the CACNA1F protein’s structure and function. According to ACMG guidelines, we therefore, believed that the hemizygous frameshift variation c.2201del (p.Val734Glyfs*17) in *CACNA1F* was more likely the pathogenic variant in family 1 with X-linked recessive CORD (PVS1 + PM2 + PP1 + PP3).

**TABLE 1:** Clinical phenotype and ERG data in six Chinese families with CORD.

Patient	Male	Age (y)	Diopter		BCVA		Fundus examination		Central retina		ERG									
			OD	OS	OD	OS	OD	OS	OD	OS	Scotopic 0.01		Scotopic 3.0				Photopic 3.0			
											b-wave (μV)		a-wave (μV)		b-wave (μV)		a-wave (μV)		b-wave (μV)	
											OD	OS	OD	OS	OD	OS	OD	OS	OD	OS
F1	Male	34	−11.00DS/–1.25DC*35°	−9.50DS/−2.25DC*175°	0.4	0.4-	Fundus tessellation	Fundus tessellation	No reflection in the macular fovea	No reflection in the macular fovea	26.1	33.2	127	145	156	165	17.3	18.3	14.7	20.4
F2	Male	7	−9.00DS/–2.00DC*180°	−9.50DS/−2.75DC*180°	0.3	0.3+	Fundus tessellation	Fundus tessellation	Atrophy	Atrophy	59	96.7	170	223	172	191	18.4	32.6	29.2	16.2
F3	Female	16	−9.00DS/−2.50DC*155°	−11.00DS/−3.75DC*10°	0.3	0.1	Fundus tessellations	Fundus tessellations	Elliptic atrophy	Elliptic atrophy	9.3	19.6	3.91	10.6	141	136	4.19	5.91	4.93	4.61
F4	Male	6	−1.25DS/−2.00DC*180°	+0.50DS/−3.50DC*175°	0.3	0.1	Macular coloboma-like lesion with a central atrophic scar surrounded by a ring of pigmentary changes	Macular coloboma-like lesion with a central atrophic scar surrounded by a ring of pigmentary changes	Coloboma-like changes	Coloboma-like changes	23.2	30	71	83.3	187	225	11.5	23.9	58.6	52.4
F5	Female	47	−0.50DS	−0.50DS/−0.50DC*35°	0.8	1.0	Elliptic atrophy lesion	Elliptic atrophy lesion	Atrophy	Atrophy	42	38.8	111	87.7	161	129	13.6	17.7	12.5	16.3
F6 II:1	Male	59	−7.00DS	−7.00DS°	1.0	1.0	Fundus tessellation	Fundus tessellation	Geographic atrophy	Geographic atrophy	25.4	39	64.3	73.7	157	169	7.33	11.1	8.83	14.5
F6 II:4	Female	51	−7.00DS/–1.25DC*45	−9.00DS/–1.75DC*150	0.02	0.1	Fundus tessellation	Fundus tessellation	Geographic atrophy	Geographic atrophy	43	51	90.1	81.2	271	242	17.9	18.4	32	26.5

**TABLE 2:** Bioinformatics and pathogenicity analyses of variants in six Chinese families with CORD.

	Proband 1	Proband 2	Proband 3	Proband 4	Proband 5	Proband 6
Gene	*CACNA1F*	*CACNA1F*	*PROM1*	*ADAM9*	*CRX*	*CRX*
Nucleotide	c.2201del	c.245G>A	c.2373+5G>T	c.604C>T	c.682C>T	c.682C>T
Amino acid	p.V734Gfs*17	p.Arg82Gln	—	p.Arg202Ter	p.Gln228Ter	p.Gln228Ter
NM_	NM_005183.4	NM_005183.4	NM_006017.3	NM_003816.3	NM_000554.6	NM_000554.6
Exon	Exon 16	Exon 2	—	Exon 6	Exon 4	Exon 4
Inheritance	Mother	Mother	Father and mother	Father and mother	Mother	Mother
1000g2015aug_all	—	—	—	—	—	—
1000g2015aug_eas	—	—	—	—	—	—
esp6500siv2_all	—	—	—	—	—	—
all_gnomAD	—	—	—	—	—	—
eas_gnomAD	—	—	—	—	—	—
SIFT_	—	D	—	—	—	—
Polyphen2_HDIV_	—	D	—	—	—	—
Polyphen2_HVAR_	—	D	—	—	—	—
LRT	—	D	—	D	D	D
MutationTaster	—	D	—	A	D	D
MutationAssessor	—	H	—	—	—	—
FATHMM_	—	T	—	—	—	—
PROVEAN_	—	D	—	—	—	—
CADD_	—	3.764	—	8.151	8.361	8.361
CADD_phred score	—	26.1	—	41	42	42
GERP++	—	5.15	—	4.64	4.21	4.21
phyloP100way_	—	7.804	—	2.196	7.109	7.109
phastCons100way_	—	1	—	1	1	1
REVEL_score	—	0.807	—	—	—	—

### 3.2 Family 2

The proband of family 2 was a 7-year-old boy who complained of progressively reduced vision in both eyes with photophobia for 1 year. He denied a family history of hereditary disease and consanguineous marriage (Figure 1 F2-A). He had dyserythrochloropsia and BCVA of 0.3 in both eyes (Table 1). The anterior segment was normal, fundus tessellation with macular atrophy was observed, and the peripheral retina did not show obvious bone-spicule pigmentation. OCT showed thinning of the outer layer of the macula and the disappearance of the light reflection signal in the ellipsoid zone and chimeric zone of fovea centralis, and autofluorescence in both eyes suggested hypofluorescence in the macula (Figure 2-F2). ERG recording showed slightly reduced scotopic and significantly diminished photopic responses (Table 1). The condition was diagnosed as CORD according to the clinical phenotype. The proband carried a hemizygous missense variant c.245G>A (p.Arg82Gln) in *CACNA1F*, and Sanger sequencing showed that the proband’s unaffected mother carried the same heterozygous variant in *CACNA1F* (Table 2), consistent with the mode of X-linked recessive inheritance. The East Asian population (ExAC_EAS) and the gnomAD databases and prior reports of the variant were negative (PM2_Moderate). This variant has been reported in the literature in congenital stationary night blindness and early detection of high myopia (Zeitz et al., 2015; Sun et al., 2015; Zeitz et al., 2019) (PS1_Strong). The substitution, c.245G>A, caused the amino acid change from arginine to glutamine at residue 82 (p.Arg82Gln), and four software predictions of SIFT, PolyPhen-2, CADD, and REVEL_score indicated the deleterious impact of this variant (PP3_Supporting). Moreover, proteome conservation analysis revealed that the amino acid at position 82 was substantially conserved across several taxa (Figure 1 F2-C), indicating that the variation at this location is more likely to have an impact on the CACNA1F protein’s structure and function. The codon arginine 82 was located in the topological domain. The missense variant c.245G>A (p.Arg82Gln) would lead to the conversion of charged residues to uncharged residues, and the interacting amino acid residues would change from S68, L75, N79, C85, V147, and A148 to T76, N79, and I86, resulting in topological changes that affect protein function (Figure 1 F2-D). According to ACMG guidelines, we therefore believed that the hemizygous missense variant c.245G>A (p.Arg82Gln) in the *CACNA1F* gene was more likely the pathogenic variant in family 2 with X-linked recessive CORD (PS1 + PM2 + PP3).

### 3.3 Family 3

The proband of family 3 was a 16-year-old girl who complained of reduced vision in both eyes for 3 years, and she had a history of consanguineous marriage of her parents (Figure 1 F3-A). She had dyserythrochloropsia in both eyes. BCVA was 0.3 in the right eye and 0.1 in the left eye (Table 1). The anterior segment was normal, fundus tessellation with elliptic atrophy lesion was observed in the macular area, and the peripheral retina did not show obvious bone-spicule pigmentation. OCT showed significant atrophy and the thinning of the outer retina in the macula, and autofluorescence suggested that hyperfluorescence lesions were surrounded by low fluorescence in the macula of both eyes (Figure 2-F3). ERG suggested a significant decrease in response amplitude for both scotopic adaptation and photopic adaptation (Table 1). The condition was diagnosed as CORD according to the clinical phenotype. The proband carried a homozygous splice variant c.2373+5G>T in *PROM1*, and both the phenotypically normal father and mother carried the same heterozygous variant at this locus as validated by Sanger sequencing (Table 2), suggesting the co-segregation of the genotype and clinical phenotype, consistent with the mode of an autosomal recessive inheritance. The variant has not been detected in the East Asian population (ExAC_EAS) and gnomAD databases. This variant has been identified in the literature in a consanguineous family member with retinitis pigmentosa (Chen et al., 2013). The proband was clinically consistent with the disease caused by this gene, and various bioinformatics computing software programs predicted that the variant affected mRNA splicing. We therefore believed that the homozygous splice variant c.2373+5G>T in *PROM1* was more likely the pathogenic variant present in family 3 with CORD.

### 3.4 Family 4

The proband of family 4 was a 6-year-old boy who complained of reduced vision in both eyes for 3 years. He denied a family history of hereditary disease and consanguineous marriage (Figure 1 F4-A). He had dyserythrochloropsia in both eyes. BCVA was 0.3 in the right eye and 0.1 in the left eye (Table 1). The anterior segment was normal and the central retina had a macular coloboma-like lesion with a central atrophic scar surrounded by a ring of pigmentary changes, including a yellowish pigmentation of both eyes. OCT showed the atrophic and ill-defined retinal layers, alterations, and disruptions of the retinal pigment epithelium and macular posterior staphyloma. Autofluorescence showed hypofluorescence in the macula of both eyes (Figure 2-F4). ERG results suggested a mild reduction in the b-wave amplitude of the photopic cone response and a severe reduction in the b-wave amplitude of the scotopic rod response in both eyes (Table 1). The condition was diagnosed as CORD according to the clinical phenotype. The proband carried a homozygous nonsense variant c.604C>T (p.Arg202Ter) in *ADAM9*, and the phenotypically normal father and mother carried the same heterozygous variant (Table 2). The variant has not been detected in the East Asian population (ExAC_ EAS) and gnomAD databases, and it has not been reported previously (PM2_Moderate). The nonsense variant, c.604C>T, was anticipated to create an early termination codon at residue 202, causing the early termination of polypeptide chain synthesis. As a result, the majority of the proteins generated were inactive or lost their usual function (PVS1_Very Strong), and various bioinformatics computing software programs indicated the deleterious impact of this variant (PP3_Supporting). It is anticipated that mutant alleles’ expression of the ADAM9 protein (including shortened polypeptides) will be eliminated via NMD. Moreover, proteome conservation analysis revealed that the amino acid at position 202 was substantially conserved across several taxa (Figure 1 F4-C), indicating that the variation at this location is more likely to have an impact on the ADAM9 protein’s structure and function. According to ACMG guidelines, we therefore believed that the homozygous nonsense variant c.604C>T (p.Arg202Ter) in *ADAM9* was more likely the pathogenic variant present in family 4 with CORD (PVS1 + PM2 + PP3).

### 3.5 Family 5

The proband of family 5 was a 47-year-old woman who complained of poor night vision in both eyes for 1 year. She denied a consanguineous marriage history, and her mother had impaired vision and night blindness for many years (Figure 1 F5-A). The proband had normal color vision, and BCVA was 0.8 in the right eye and 1.0 in the left eye (Table 1). Elliptic atrophy lesion was observed in the retinal region with no foveal reflection, and the anterior segment was normal. OCT showed atrophy of the outer retina and RPE layer in the macula, and autofluorescence suggested that hyperfluorescence lesions were surrounded by low fluorescence in the macula of both eyes (Figure 2-F5). ERG recording revealed a moderate decline in scotopic and severe or moderately reduced photopic responses (Table 1). The condition was diagnosed as CORD according to the clinical phenotype. The proband carried the heterozygous nonsense variant c.682C>T (p.Gln228Ter) in *CRX* (Figure 1 F5-B Table 2). This variant has not been previously reported and was also not detected in the East Asian population (ExAC_EAS) and gnomAD databases (PM2_Moderate). This variant has been reported in the literature in a retinitis pimentosa patient (Ge et al., 2015) (PP5_Supporting). The nonsense variant, c.682C>T, was predicted to generate a premature termination codon at residue 228, which would result in a premature termination of polypeptide chain synthesis, and most of the proteins produced were inactive or lost their normal function (PVS1_Very Strong), and various bioinformatics computing software programs indicated the deleterious impact of this variant (PP3_Supporting). The nonsense variation was predicted to cause protein truncation length >10% but not nonsense-mediated mRNA degradation because it was positioned in the final exon. Moreover, proteome conservation analysis revealed that the amino acid at position 228 was substantially conserved across several taxa (Figure 1 F5-C), indicating that the variation at this location is more likely to have an impact on the CRX protein’s structure and function. According to ACMG guidelines, we therefore believed that the heterozygous nonsense variant c.682C>T (p.Gln228Ter) in *CRX* was more likely the pathogenic variant observed in family 4 with CORD (PVS1 + PM2 + PP3 + PP5).

### 3.6 Family 6

The proband of family 6 was a 59-year-old man who complained of reduced vision in both eyes for 3 years. He denied a consanguineous marriage history, and his younger sister had severe vision loss (Figure 1 F6-A). His color vision was normal, and both of his eyes had a BCVA of 1.0 (Table 1). The anterior segment was normal, and fundus tessellation with geographic atrophy was observed in the macula. OCT showed obvious thinning of central retinal thickness, and the outer nuclear layer and the ellipsoid zone disappeared in both eyes. Autofluorescence showed hypofluorescence lesions in the macula of both eyes (Figure 2-F6 Ⅱ:1). A slight fall in scotopic and a sharp reduction in photopic responses were detected in ERG recordings (Table 1). His younger sister had a normal color vision, and BCVA was 0.02 in the right eye and 0.1 in the left eye (Table 1). The anterior segment was normal, and fundus tessellation with macular atrophy was observed. OCT showed atrophy of the outer retina and RPE layer in the macula, and autofluorescence suggested that hyperfluorescence lesions were surrounded by low fluorescence in the macula of both eyes (Figure 2-F6 II:4). ERG recording revealed a moderate decline in scotopic and severe or moderately reduced photopic responses (Table 1). The condition was diagnosed as CORD in both patients according to the clinical phenotype. The proband as well as his younger sister carried the heterozygous nonsense variant c.682C>T (p.Gln228Ter) in *CRX* (Table 2). Moreover, proteome conservation analysis revealed that the amino acid at position 228 was substantially conserved across several taxa (Figure 1 F6-C), indicating that the variation at this location is more likely to have an impact on the CRX protein’s structure and function.

## 4 Discussion

X-linked cone–rod dystrophy (CORDX) is characterized by genetic variations found in three specific loci on the X chromosome. Particularly, CORDX1 is caused by variants in *RPGR* (Xp21.1), CORDX2 is located at Xq27.2−28, and CORDX3 is caused by variants in *CACNA1F* (Xp11.23) (Jalkanen et al., 2003b; Zeitz et al., 2015). The voltage-dependent calcium channel α1F subunit (*CACNA1F*) gene, located on human chromosome Xp11.23, contains 48 exons and encodes a retina-specific expression of the Cav1.4 ion channel α1F subunit consisting of 1,977 amino acids (McRory et al., 2004; McRory et al., 2004). Cav1.4 is typically observed in the outer plexiform, inner nuclear, inner plexiform, and nerve fiber layers of the retina (McRory et al., 2004). Furthermore, it is also localized at the distal regions of optic rods and cone cells and facilitates the exocytosis of neurotransmitters through the ribbon synapse of retinal photoreceptors, hence exerting a crucial function in the transmission of signals from photoreceptors to secondary retinal neurons (Morgans, 2001). *CACNA1F* is the first gene found on the X chromosome to cause congenital stationary night blindness (CSNB) (Bech-Hansen et al., 1998). The identification of a splicing variant in the *CACNA1F* gene was initially reported in a family affected with CSNB in Finland in the year 2006 (Jalkanen et al., 2006), confirming the association of such gene with the occurrence of CORDX. Subsequent reports have successively confirmed the association of *CACNA1F* with the occurrence of CORDX (Hauke et al., 2013). In the present investigation, it was shown that individuals harboring the *CACNA1F* variation exhibited fundus tessellation, concomitant with irregularities in the outer macular layer. Nevertheless, it is worth noting that the patient harboring the nonsensical variant exhibited an earlier onset of symptoms and a decline in visual acuity, potentially indicating an association with genetic variability. The variant locus c.245G>A (p.Arg82Gln) in *CACNA1F* has been previously reported in families with CSNB (Zeitz et al., 2015), but the clinical phenotype was not described in detail, whereas the patient carrying the same variant in this study had progressive vision loss as the chief complaint, and the patient did not complain of night blindness, and as questioned repeatedly, we diagnosed the patient with CORDX in combination with the mild decrease in the amplitudes of b-wave of scotopic 0.01 ERG and a- and b-waves of 3.0 ERG and the severe decrease in the amplitudes of a- and b-wave of photopic 3.0 ERG. Additionally, some studies found that *CACNA1F* is closely associated with the onset of Aland Island eye disease (Jalkanen et al., 2007), characterized by fundus pigmentation, reduced visual acuity, nystagmus, astigmatism, color vision defects, and dark maladjustment. Thus, it is evident that there is a significant overlap in the clinical phenotypes of the three diseases caused by *CACNA1F*. Hence, conducting a thorough and extensive clinical examination and analysis can serve to elucidate the diagnosis and mitigate the occurrence of misinterpretation and overlooked diagnoses in clinical settings, alongside the utilization of genetic testing.

PROM1 is a transmembrane glycoprotein originally thought to be a biomarker for human hematopoietic stem and progenitor cells (Miraglia et al., 1997) and in the retina. *PROM1* is mainly localized in the outer segment of retinal photoreceptors and plays a key role in the membranous disc morphogenesis of the photoreceptor outer segment (Yang et al., 2008). Some studies have shown that in CORD, approximately 1%–9.5% of cases are caused by the *PROM1* variant (Littink et al., 2010; Boulanger-Scemama et al., 2015; Birtel et al., 2018b). Overall, the *PROM1* variant is associated with multiple diseases with overlapping phenotypes, such as retinitis pigmentosa, cone–rod dystrophy, Stargardt-like macular dystrophy, and bull’s eye macular dystrophy (Littink et al., 2010; Boulanger-Scemama et al., 2015; Birtel et al., 2018b; Michaelides et al., 2010). Depending on the variant, the age of the onset, first symptoms, and the severity of the disease vary among patients. The *PROM1* variant can present both recessive and dominant genetic inheritance (Boulanger-Scemama et al., 2015). Compared with retinal dystrophy caused by the dominantly inherited *PROM1* variant, patients with retinal dystrophy associated with recessive inherited *PROM1* variants, such as CORD and Leber congenital amaurosis, have an early onset and more severe clinical phenotype and can present high myopia and vision loss at the early stage of the disease (Michaelides et al., 2010; Eidinger et al., 2015; Cehajic-Kapetanovic et al., 2019), which is highly consistent with the clinical phenotype of the patient in this study. The majority of frameshift and nonsense variations caused premature termination codons, which hastens the nonsense-mediated decay of shortened modified RNAs before translation (Maw et al., 2000). Missense and splice variants associated with recessive disease may cause null (or near null loss of function) effects, as evident from similar phenotypes of patients with homozygote missense and splice variants and patients with truncating variants of similar age, and these loss-of-function (LOF) variants are associated with optic disc membrane disorders and photoreceptor degeneration (Zacchigna et al., 2009), which can lead to disease. The homozygous variant c.2373+5G>T in *PROM1* detected in this study was previously detected in an RP patient with a consanguineous family history, but the clinical phenotype was unknown (Chen et al., 2013). The parents of the patient in this study were also consanguineous, and the patient complained of reduced vision in both eyes. Clinically, CORD patients need to be differentiated from RP when they show significant rod dysfunction in the late stages often accompanied by night blindness symptoms. Typical RP usually starts with night blindness as the first symptom, often not accompanied by photophobia and high myopia. In this study, fundus tessellation with an elliptic atrophy lesion was observed in the macular area of the patient carrying the *PROM1* variant. The lesions showed hypofluorescence changes in autofluorescence, and there was no typical RP fundus appearance. Repeatedly questioning the medical history, the patient had no night blindness, and the ERG results suggested severe dysfunction of both cone and rod cells. Based on the patient’s chief complaint, clinical phenotype, and ERG examination, this patient was finally diagnosed with CORD.

*ADAM9* is located on chromosome 8 and contains 22 exons, which are involved in a number of biological processes, such as cell division, proteolysis, cell adhesion, cell fusion, and signal transmission, which encode membrane proteins (Mahimkar et al., 2005). The brain, spinal cord, retina, and lens all express *ADAM9*, which participates in a variety of pathways including extracellular matrix interactions (Schwettmann and Tschesche, 2001). It primarily functions in the link between RPE and photoreceptor outer segments in the human retina (Seals and Courtneidge, 2003). Parry et al. (2009) first reported the *ADAM9* variant causing CORD in four consanguineously married families with poor vision before the age of 10 years without nystagmus and photophobia. Atrophy of the outer layer of the retina was observed in the macula area of the fundus. Scattered white patches were observed in the posterior pole and optic disc in most patients, which may be accompanied by pre-equatorial pigmentosa retinopathy in older patients. CORD caused by the *ADAM9* variant as reported so far were all from consanguineously married families, and the type of variant was a homozygous splice or nonsense variant, all of which could produce termination codon. It was found from the studies that the pathogenic mechanism of *ADAM9* is LOF, and the early occurrence of the termination codon can lead to nonsense-mediated mRNA degradation, which leads to the onset of the disease (Parry et al., 2009). The family of this study denied a consanguineous marriage history, the onset age was young, and the disease was severe, which was consistent with pathogenesis characteristics reported in previous studies. However, there was no significant characteristic of fundus lesions caused by this gene variant (Hull et al., 2015). Since CORD studies related to the *ADAM9* variant were all case reports and lacked large sample studies, the relationship between the genotype and clinical phenotype cannot be determined yet and needs further exploration.

*CRX*, located on chromosome 19q13.33, is a cone–rod isoform gene (OMIM: 602225). It encodes a 299-amino acid isoform transcription factor necessary for photoreceptor development and survival and is highly similar to the OTX family isoform gene (Freund et al., 1997). Animal studies have shown that *CRX* is most prominently expressed in the vertebrate retina’s photoreceptor cells and pineal cells (Chen et al., 1997) and plays a key role in the differentiation and maintenance of photoreceptor cells by interacting synergistically with other transcription factors, such as the neural retina-specific leucine zipper protein (NRL), retina and anterior neural fold homeobox protein (RAX), and nuclear receptor subfamily 2 group E member 3 (NR2E3) (Peng et al., 2005). *CRX* variants can lead to not only dominant CORD but also dominant retinitis pigmentosa and Leber congenital amaurosis (Sohocki et al., 2001; Freund et al., 1998). CRX proteins have five conserved functional domains, namely, the homeodomain (HD) (from N-terminal to C-terminal), glutamine-rich (Gln) domain, infrastructure domain, WSP domain, and OTX-tail domain. The homeodomain is associated with DNA binding, while the C-terminal region of the protein is associated with transcriptional regulation (Chau et al., 2000). It was found from the studies that the missense variants in *CRX* occur in the homeodomain, while frameshift and nonsense variants are confined to the C-terminal region of the protein (Tran and Chen, 2014).

In this study, the nonsense and frameshift variations in four families with CORD generate premature stop codons and C-terminal truncation variations in *CACNA1F*, *ADAM9*, and *CRX*. They might introduce premature translational-termination codons (PTCs) that NMD can detect and degrade. NMD functions as a widespread RNA surveillance system for mRNA quality in eukaryotic cells. By stopping the translation of the aberrant mRNA and changing the phenotype, it can identify mutant mRNAs carrying PTCs and quickly degrade and remove aberrant transcripts to prevent the buildup of shortened and potentially dangerous proteins. PTCs situated upstream of the last exon junction complex (EJC) make NMD effective (Khajavi et al., 2006). Many rules that were consistent with our findings govern this efficiency. The final exon rule and the 50-nt rule are considered to be canonical rules (Nagy and Maquat, 1998; Le Hir et al., 2001; Holbrook et al., 2004). The 50-nt rule states that PTCs that are fewer than 50 nucleotides (nt) upstream of the final exon–exon junction will cause NMD, whereas PTCs that are located in the final exon of a gene or in the final 50 bases of the penultimate exon will not cause NMD (the last exon rule). Family 1 in our study involved a variation, p.Val734Glyfs*17, located on exon 6 in *CACNA1.* Consistent with the 50-nt rule, the physical sites of this variant were not on the last or the penultimate exon as NMD is believed to play a role in the phenotypes, presenting a mild clinical phenotype. Families 5 and 6 in our study involved the same variation, p.Gln228Ter, located on exon 4 in *CRX.* Consistent with the last exon rule, the physical site of this variant was on the last exon as NMD is not triggered to play a role in the phenotypes (the last exon rule), presenting severe clinical phenotypes (Conti and Izaurralde, 2005; Inoue et al., 2004; Kerr et al., 2001).

However, sometimes NMD does not absolutely degrade all transcripts containing PTCs, and some truncated mutant proteins are likely to be expressed, which leads to a more severe clinical phenotype. In this study, family 4 involved a variation, p.Arg202Ter, located on exon 6 in *ADAM9.* Consistent with the 50-nt rule, the physical sites of this variant were not on the last or the penultimate exon as NMD might be triggered to play a role in the phenotypes, presenting a mild clinical phenotype. However, because NMD fails to completely degrade all transcripts containing PTCs, some truncated mutant proteins are likely to be expressed, resulting in severe visual impairment and macular degeneration of the patient phenotype. In addition, it is likely that NMD is not the only pathway that is actually responsible for mRNA stability/degradation, thus modulating disease severity (Khajavi et al., 2005; Chan et al., 1998).

It is worth noting that the cases of patients in the two CORDX families, one AR CORD family, and one AD CORD family in this study were complicated with high myopia. It was found from the studies that approximately 39% of pathogenic variants in people with high myopia are found in genes associated with retinal inherited disease (Haarman et al., 2022), and many patients with retinal dystrophy often present high myopia or even early-onset high myopia in the early stages, while symptoms associated with retinal dystrophies such as dyschromatopsia and night blindness are not prominent. Therefore, these disorders are easily overlooked and often result in misdiagnosis and missed diagnosis. Two investigations of children with high myopia conducted in the community have further supported this (Logan et al., 2004; Marr et al., 2001). In light of this, we advise clinicians to deeply interrogate patients with high myopia about their medical history and family history in order to identify a particular genetic pattern. This should be followed by a detailed ophthalmologic examination and the necessary systemic examination to look for clinical signs other than myopia, and if the patient is suspected of having a Mendelian disorder, whole-genome exome sequencing can be performed to further clarify the diagnosis.

In conclusion, our study successfully identified three previously unreported genetic variations in CORD genes across six distinct CORD families, each exhibiting unique inheritance patterns. Our study was accomplished through the utilization of WES) and Sanger sequencing techniques. This discovery extends the range of pathogenic variants in CORD genes, hence offering valuable insights into genetic counseling in families.

## Data Availability

The data that support the findings of this study are openly available in the GenBank repository (https://www.ncbi.nlm.nih.gov/genbank/). Accession numbers: 2634202, 2634179, 2634114, 2634096.
